# Ongoing rabies outbreak in dogs of unprecedented scale and human cases in Nelson Mandela Bay Municipality, South Africa, up to 13 February 2022

**DOI:** 10.2807/1560-7917.ES.2022.27.16.2200252

**Published:** 2022-04-21

**Authors:** Matthijs F Ravensberg, Ewout B Fanoy, Jane Whelan, Carmen WE Embregts, Corine H GeurtsvanKessel, Jared B Strydom

**Affiliations:** 1 Veterinarian, Private sector, Port Elizabeth, South Africa; 2 Infectious disease department, Public Health Service Rotterdam-Rijnmond, Rotterdam, the Netherlands; 3 Department of Viroscience, Erasmus Medical Centre, Rotterdam, the Netherlands; 4 State Veterinarian Animal Health, Department: Rural development and Agrarian Reform, Port Elizabeth, South Africa

**Keywords:** rabies, lyssavirus, disease outbreaks, dogs, animals, South Africa

## Abstract

More than 430 cases of rabies have been confirmed in dogs in the Nelson Mandela Bay Metropolitan Municipality of South Africa since July 2021. We describe the ongoing outbreak, its geographical spread and six related human deaths that have occurred. Further investigation of the outbreak and vaccination of the dog population is required. Raising awareness among healthcare providers, the public, and among international travellers planning to visit the region, is key for action to protect human and animal health.

The Nelson Mandela Bay Metropolitan Municipality (NMBM) in the Eastern Cape Province of South Africa is currently experiencing a large outbreak of rabies in the dog population. Since July 2021, over 430 canine cases and six related human deaths have been confirmed in the metropolitan area. Here, we describe the canine outbreak and related human cases as part of an ongoing investigation, and our aim is to raise awareness among local citizens and visitors to the region in order to prevent onward transmission to human hosts.

## Rabies surveillance in South Africa

Rabies in animals is a notifiable disease in South Africa, according to the Animal Diseases Act - Number 35 of 1984 [1]. A case of rabies is usually suspected based on changes in animal behaviour, i.e. displaying signs of neurological disease. Once suspected, cases are reported to the responsible state veterinarian by state and private veterinarians, welfare organisations and the municipal dog control unit [2]. Dogs with suspected rabies that do not die from the disease are euthanised.

Data on suspected and confirmed animal cases are currently being collected and reported by the Department of Agriculture Land Reform and Rural development (DALRRD). Human rabies cases are also notifiable, and are reported under separate legislation to The National Institute for Communicable Diseases (NICD) [3]. For this report, we compiled data on laboratory-confirmed human and canine cases, and constructed an epidemiological curve. A map was created using a template from https://www.openstreetmap.org.

## Identification and evolution of the outbreak

**Figure 1:**
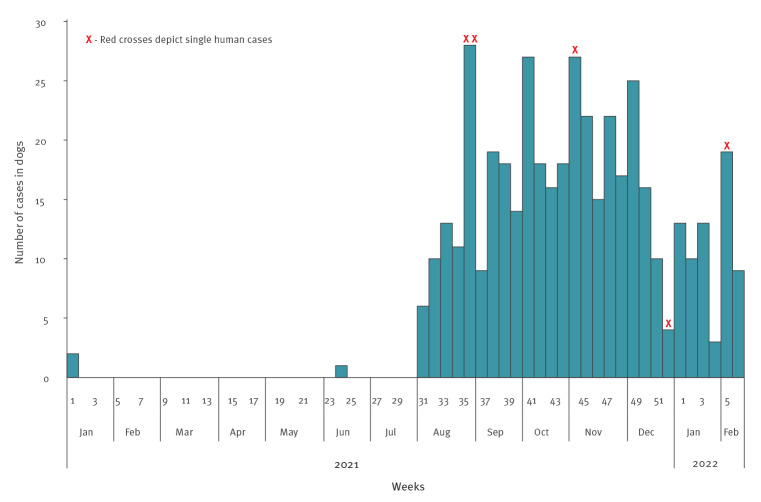
Epidemiological curve of weekly cases of rabies in dogs (n = 436) and date of exposure in humans (n = 5) in the Nelson Mandela Bay Municipality, South Africa, 1 January 2021–13 February 2022

**Figure 2:**
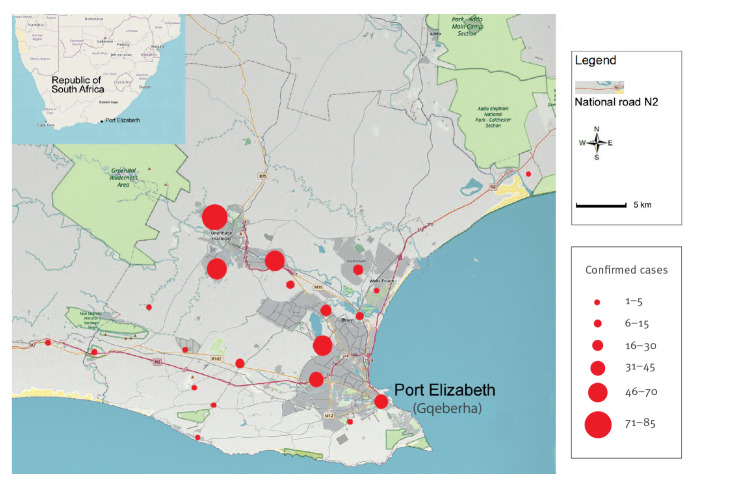
Geographical distribution of rabies cases in dogs in the Nelson Mandela Bay Municipality, South Africa, 1 January 2021–13 February 2022 (n = 436)

On 8 January 2021, a domestic and a stray dog in the eastern part of the NMBM in Bluewater Bay tested positive for rabies. By the end of July, reports on confirmed rabid dogs were beginning to increase and rabies virus was spreading within the canine population. Of specimens submitted and analysed in September 2021, 74% (64/86) tested positive. By early February 2022, there were 436 confirmed cases in dogs (Figure 1) detected throughout the municipality (Figure 2), an area with a population of 1.26 million people [4] encompassing the city of Port Elizabeth (official name: Gqeberha) and the towns Despatch and Uitenhage.

Of laboratory-confirmed canine cases, 69% of samples were collected through animal welfare organisations, 27% through private veterinarians and 4% directly by the state veterinarian. Cases in dogs are still forthcoming. Human cases for whom a date of exposure was known (n = 5/6) are denoted (X); all human cases have since died.

Clinical signs of rabies noted in dogs were typically neurological, such as salivation, protruding tongue, inability to swallow, ataxia and paresis of the hind legs. Behavioural changes included a change in temperament, aggression or lethargy. Vaccination status of the dog is requested on the submission form but is rarely known or completed [5]. The breed, whether stray or domestic, or clinical outcomes are also not routinely reported on the submission form. All dogs with confirmed rabies, however, either died naturally as the disease progressed or were euthanised (personal communication: J Strydom, 7 March 2022).

Of the human cases, there were six confirmed exposures during the study period, all of whom have since died: one adult (aged 60–69 years), one adolescent (aged 10–19 years) and four children (aged 0–9 years). Fifty percent were female. All cases involved a dog bite: three cases were bitten on the hand or arm, two cases on the head or face, and one case on both arm and face. Date of exposure was known for 5 of 6 cases (Figure 1). Notably, we did not have access to individual clinical or treatment data on human cases.

## Control measures taken

To control the outbreak, the Eastern Cape Department: Rural Development and Agrarian Reform (E.C. DRDAR) started free, mass rabies vaccination campaigns among dogs and cats throughout affected areas in August 2021, and a roster was advertised publicly with dates and locations. In September, local media coverage promoted the importance of vaccination and early intervention to manage dog bites [6]. Private veterinarians were also offering free or reduced cost vaccinations and participating in rabies vaccination campaigns with stock provided by the E.C. DRDAR and the South African Veterinary Association (SAVA), though provision of free vaccines gradually ceased towards the end of 2021. Since January 2021, ca 60,000 parenteral rabies vaccinations have been administered, the majority to dogs.

## Specimen collection and education of animal and human healthcare professionals

To facilitate specimen collection from animals with suspected rabies, euthanasia should be performed in a way that leaves the brain intact. The state veterinarian will then send a carcass, whole head or brain tissue, accompanied by a standard ‘rabies submission and laboratory test report’ [5], to one of the state-approved laboratories for diagnostic purposes, e.g. Agriculture Research Council – Onderstepoort Veterinary Institute (ARC-OVI) and Allerton Provincial Veterinary Laboratory. Specimens can be frozen, but ideally transported on ice in a 50% glycerol saline solution. A suspected animal case is laboratory-confirmed using a fluorescent antibody test (FAT) on a brain smear [2,7,8].

Initially, due to lack of personal protective equipment (PPE) and correct packaging and facilities, whole dogs’ heads were sent on ice, rather than the preferred on-site collection of brain specimens using the ‘straw sample technique’ [3]. Animal health professionals would benefit from further education to safely restrain and sedate dogs and to effectively collect specimens and will require more PPE and vaccinations. The medical profession should also be alerted to consider rabies in the differential diagnosis of a possible human case. Doctors must be aware of the existing protocol for timely delivery of post-exposure prophylaxis, as it can save lives when given correctly.

## Public awareness and dog vaccination campaigns are key to human rabies prevention

The outbreak in dogs has spread rapidly both geographically – westwards with numbers increasing in the suburbs – and in terms of the overall numbers of dogs affected, reflecting the virus’s ability to penetrate susceptible dog populations. Some townships have a low incidence, but whether this is due to vaccination or under-reporting is unclear. Knowledge of the demographic structure of the dog population would help to plan and monitor effective rabies vaccination programmes and requires further study. Of an estimated 57,000 to 160,000 dogs in the region [9], at least 70% should be vaccinated to bring the reproduction number (R0) below 1 [10]. This will require additional resources for public information campaigns to raise disease awareness, to encourage vaccination of dogs, and inform about the need to seek urgent medical attention after a dog bite to prevent more human fatalities.

## Identification of the causative strain

The rabies virus strain involved in this outbreak is still unknown. It might be a continuation of the evolving epidemic of the viral strain circulating in the province of KwaZulu-Natal and the eastern, rural parts of the Eastern Cape [11,12]. There is a risk that it could progress further into the densely populated Garden Route and Cape Town, where four cases of canine rabies were already confirmed in August 2021 [13]. Genetic sequencing of human and canine specimens is needed to determine the origin of the outbreak and to explain the rapid spread among dogs. Specimens are currently sent for diagnostic purposes to one of two accredited state laboratories in South Africa. Accreditation of a nearby, local laboratory will facilitate testing and improve surveillance [14]. State facilities are also needed to isolate suspected animals, collect and store specimens.

## Discussion

Rabies is one of the oldest known zoonotic diseases. It is endemic in various animal species in South Africa, but the domestic dog is the most likely source of human rabies [10]. From 2008 to 2018, an average of 10 human cases were reported nationwide annually, and in 2021, 19 human cases were confirmed [15]. Rabies has been confirmed only occasionally in the past in NMBM in dogs or wildlife, but no rabies was confirmed in dogs in 8 years prior to this outbreak (personal communication: J Strydom, 6 April 2022) and a human case has never been confirmed in the region (personal communication: V Msimang, 11 March 2022). The distribution of human rabies cases is known to overlap with the distribution of canine cases [8] and – as humans and dogs often live in close proximity – a rabies outbreak among dogs poses an increased risk to human public health. Awareness is key then, for dog owners to have their dog vaccinated and for the local health system to manage dog bites and suspected human rabies cases, and to correctly deliver timely post-exposure prophylaxis.

Up to 13 February 2022, there are early signs that the outbreak is stabilising but anecdotally, some dogs are being euthanised based on presumptive diagnoses without samples taken for laboratory confirmation; the true scale of the outbreak is probably underestimated. Indeed, underdiagnosis and under-reporting may also be a problem. Reports of increased canine deaths were received by welfare organisations earlier in 2021 but these were initially attributed to distemper, a canine infectious disease caused by a paramyxovirus, which causes similar clinical symptoms, namely pyrexia, gastrointestinal and neurological signs. These could in fact have been undiagnosed rabies cases and possibly the outbreak evolved earlier (personal communication: J Strydom, 21 Oct 2021).

## Conclusions

The scale of this ongoing outbreak of rabies in dogs in NMBM is unprecedented, and further investigation and control measures are necessary to protect both human and animal health. Port Elizabeth is on South Africa’s ‘Garden Route’, a highly popular tourist destination. In line with current guidance, travellers to the region should be advised to avoid contact with dogs, to seek medical advice if bitten or scratched by a dog or other mammal while in South Africa. Additionally, for travellers who plan a prolonged stay, or who will potentially have contact with dogs or wildlife, rabies vaccination should be considered before arrival.
